# Draft Genome Sequence of the Intimin-Positive Enteropathogenic Escherichia albertii Strain MBT-EA1, Isolated from Lettuce

**DOI:** 10.1128/genomeA.00255-18

**Published:** 2018-04-12

**Authors:** Gregor Fiedler, Erik Brinks, Christina Böhnlein, Gyu-Sung Cho, Sabrina Koberg, Jan Kabisch, Charles M. A. P. Franz

**Affiliations:** a Department of Microbiology and Biotechnology, Max Rubner-Institut, Federal Research Institute of Nutrition and Food, Kiel, Germany

## Abstract

The genome of the intimin (*eae*)-harboring Escherichia albertii strain MBT-EA1, isolated from lettuce in Germany, was sequenced. Sequence analysis showed the assembled draft genome size to be 4,560,948 bp, containing a predicted total of 4,414 protein-encoding genes, 11 rRNAs, and 82 tRNAs. Furthermore, three plasmid sequences were found.

## GENOME ANNOUNCEMENT

Escherichia albertii is a zoonotic potential foodborne pathogen which has been isolated in various countries worldwide (1–4). In humans, E. albertii has the potential to cause severe infections (e.g., gastroenteritis), as it may integrate Shiga toxin-carrying bacteriophages into the chromosome (5, 6). The clinical significance and epidemiology of E. albertii infections are still unclear, as the microorganism was classified as a new Escherichia species in 2003 (1). Furthermore, the biochemical properties of this species are very similar to those of enteropathogenic E. coli (EPEC), which complicates their discrimination in routine diagnostics (2, 7).

We isolated an *eae*-positive E. albertii strain, MBT-EA1, from lettuce in the cold-storage area of a ready-to-eat salad-producing company. The colony showed white color on tryptone bile X-glucuronide (TBX) agar. The genomic DNA was extracted using the ZR-Fungal/Bacterial DNA-MiniPrep kit (Zymo Research, Freiburg, Germany) and quantified using a Qubit 3 fluorometer (Invitrogen, Darmstadt, Germany). The Nextera XT kit and Nextera mate-pair library prep kit were used to create libraries, and sequencing was performed as paired-end reads (2 × 251 bp) on an Illumina MiSeq platform (Illumina, Munich, Germany), according to the manufacturer’s protocols. Adapter trimming of raw reads was done using the Illumina Pipeline. Furthermore, the reads of the Nextera mate-pair library were preprocessed by AdapterRemoval (8) to remove junction adapters. The results of both runs were *de novo* assembled using SPAdes 3.09.1 (9).

The draft genome assembly consisted of 10 contigs >1,000 bp, and the *N*_50_ value was 840,915 bp. Seven contigs mapped against reference strain Escherichia albertii strain KF1 (GenBank accession number CP007025) (10) using the Mauve plugin 1.1.1 (11). The draft genome size was 4,560,948 bp, with a mol% G+C content of 49.7%. Three contigs which did not map were identified as plasmid sequences and described here as pEA1 (116,103 bp, IncFIB), pEA2 (89,048 bp, IncFII, and colicins B and M), and pEA3 [circular 3,598 bp, Col(RNAi), similar to Salmonella plasmid (GenBank accession number CP006052)]. Genomic features were identified and annotated using the NCBI Prokaryotic Annotation Pipeline (PGAP) and Rapid Annotations using Subsystems Technology (RAST) (12). The assembled genome contained 4,414 predicted genes, 82 tRNA genes, 7 5S rRNA genes, 1 16S rRNA gene, and 3 23S rRNA genes.

The sequences were analyzed *in silico* for serotype (13), *fimH* type, virulence factors (14), plasmids (15), and antibiotic resistance genes (16) using the Center for Genomic Epidemiology online service (https://cge.cbs.dtu.dk/services/) (17). The strain was identified as belonging to serotype O144:H- with the *fimH* type 461. The virulence genes found included *eaeA* (intimin), *cdtB* (cytolethal distending toxin B), *cba* (colicin B), *cma* (colicin M), *espA* (type III secretion system), *espB* (secreted protein B), *espF* (type III secretion system), *gad* (glutamate decarboxylase), and *nleB* (non-locus of enterocyte effacement [non-LEE] encoded effector). No acquired antibiotic resistance genes were found. Thus, the genome data showed that the strain, which was isolated for the first time from raw produce, may be highly virulent. Therefore, E. albertii should also be taken into consideration in safety investigations of vegetable products (18).

### Accession number(s).

This draft genome sequence of E. albertii MBT-EA1 has been deposited at DDBJ/ENA/GenBank under the accession number PTLP00000000. The version described in this paper is version PTLP01000000.

## References

[1] Huys G, Cnockaert M, Janda JM, Swings J 2003 *Escherichia albertii* sp. nov., a diarrhoeagenic species isolated from stool specimens of Bangladeshi children. Int J Syst Evol Microbiol 53:807–810. doi:10.1099/ijs.0.02475-0. 12807204

[2] Oaks JL, Besser TE, Walk ST, Gordon DM, Beckmen KB, Burek KA, Haldorson GJ, Bradway DS, Ouellette L, Rurangirwa FR, Davis MA, Dobbin G, Whittam TS 2010 *Escherichia albertii* in wild and domestic birds. Emerg Infect Dis 16:638–646. doi:10.3201/eid1604.090695. 20350378 PMC3321939

[3] Ooka T, Seto K, Kawano K, Kobayashi H, Etoh Y, Ichihara S, Kaneko A, Isobe J, Yamaguchi K, Horikawa K, Gomes TAT, Linden A, Bardiau M, Mainil JG, Beutin L, Ogura Y, Hayashi T 2012 Clinical significance of *Escherichia albertii*. Emerg Infect Dis 18:488–492. doi:10.3201/eid1803.111401. 22377117 PMC3309589

[4] Grillová L, Sedláček I, Páchníková G, Staňková E, Švec P, Holochová P, Micenková L, Bosák J, Slaninová I, Šmajs D 2017 Characterization of four *Escherichia albertii* isolates collected from animals living in Antarctica and Patagonia. J Vet Med Sci 80:138–146. doi:10.1292/jvms.17-0492. 29249728 PMC5797873

[5] Brandal LT, Tunsjø HS, Ranheim TE, Løbersli I, Lange H, Wester AL 2015 Shiga toxin 2a in *Escherichia albertii*. J Clin Microbiol 53:1454–1455. doi:10.1128/JCM.03378-14. 25653403 PMC4365202

[6] Ooka T, Ogura Y, Katsura K, Seto K, Kobayashi H, Kawano K, Tokuoka E, Furukawa M, Harada S, Yoshino S, Seto J, Ikeda T, Yamaguchi K, Murase K, Gotoh Y, Imuta N, Nishi J, Gomes TA, Beutin L, Hayashi T 2015 Defining the genome features of *Escherichia albertii*, an emerging enteropathogen closely related to *Escherichia coli*. Genome Biol Evol 7:3170–3179. doi:10.1093/gbe/evv211. 26537224 PMC4700944

[7] Abbott SL, O'Connor J, Robin T, Zimmer BL, Janda JM 2003 Biochemical properties of a newly described *Escherichia* species, *Escherichia albertii*. J Clin Microbiol 41:4852–4854. doi:10.1128/JCM.41.10.4852-4854.2003. 14532241 PMC254372

[8] Schubert M, Lindgreen S, Orlando L 2016 AdapterRemoval v2: rapid adapter trimming, identification, and read merging. BMC Res Notes 9:88. doi:10.1186/s13104-016-1900-2. 26868221 PMC4751634

[9] Nurk S, Bankevich A, Antipov D, Gurevich AA, Korobeynikov A, Lapidus A, Prjibelski AD, Pyshkin A, Sirotkin A, Sirotkin Y, Stepanauskas R, Clingenpeel SR, Woyke T, McLean JS, Lasken R, Tesler G, Alekseyev MA, Pevzner PA 2013 Assembling single-cell genomes and mini-metagenomes from chimeric MDA products. J Comput Biol 20:714–737. doi:10.1089/cmb.2013.0084. 24093227 PMC3791033

[10] Fiedoruk K, Daniluk T, Swiecicka I, Murawska E, Sciepuk M, Leszczynska K 2014 First complete genome sequence of *Escherichia albertii* strain KF1, a new potential human enteric pathogen. Genome Announc 2:e00004-14. doi:10.1128/genomeA.00004-14. 24482506 PMC3907721

[11] Darling AE, Mau B, Perna NT 2010 progressiveMauve: multiple genome alignment with gene gain, loss and rearrangement. PLoS One 5:e11147. doi:10.1371/journal.pone.0011147. 20593022 PMC2892488

[12] Overbeek R, Olson R, Pusch GD, Olsen GJ, Davis JJ, Disz T, Edwards RA, Gerdes S, Parrello B, Shukla M, Vonstein V, Wattam AR, Xia F, Stevens R 2014 The SEED and the rapid annotation of microbial genomes using subsystems technology (RAST). Nucleic Acids Res 42:D206–D214. doi:10.1093/nar/gkt1226. 24293654 PMC3965101

[13] Joensen KG, Tetzschner AMM, Iguchi A, Aarestrup FM, Scheutz F 2015 Rapid and easy *in silico* serotyping of *Escherichia coli* isolates by use of whole-genome sequencing data. J Clin Microbiol 53:2410–2426. doi:10.1128/JCM.00008-15. 25972421 PMC4508402

[14] Joensen KG, Scheutz F, Lund O, Hasman H, Kaas RS, Nielsen EM, Aarestrup FM 2014 Real-time whole-genome sequencing for routine typing, surveillance, and outbreak detection of verotoxigenic *Escherichia coli*. J Clin Microbiol 52:1501–1510. doi:10.1128/JCM.03617-13. 24574290 PMC3993690

[15] Carattoli A, Zankari E, García-Fernández A, Voldby Larsen M, Lund O, Villa L, Møller Aarestrup F, Hasman H 2014 *In silico* detection and typing of plasmids using PlasmidFinder and plasmid multilocus sequence typing. Antimicrob Agents Chemother 58:3895–3903. doi:10.1128/AAC.02412-14. 24777092 PMC4068535

[16] Zankari E, Hasman H, Cosentino S, Vestergaard M, Rasmussen S, Lund O, Aarestrup FM, Larsen MV 2012 Identification of acquired antimicrobial resistance genes. J Antimicrob Chemother 67:2640–2644. doi:10.1093/jac/dks261. 22782487 PMC3468078

[17] Thomsen MCF, Ahrenfeldt J, Cisneros JLB, Jurtz V, Larsen MV, Hasman H, Aarestrup FM, Lund O 2016 A bacterial analysis platform: an integrated system for analysing bacterial whole genome sequencing data for clinical diagnostics and surveillance. PLoS One 11:e0157718. doi:10.1371/journal.pone.0157718. 27327771 PMC4915688

[18] Sharma M, Kniel KE, Derevianko A, Ling J, Bhagwat AA 2007 Sensitivity of *Escherichia albertii*, a potential food-borne pathogen, to food preservation treatments. Appl Environ Microbiol 73:4351–4353. doi:10.1128/AEM.03001-06. 17468283 PMC1932773

